# *Cryptochrome* Regulates Circadian Locomotor Rhythms in the Small Brown Planthopper *Laodelphax striatellus* (Fallén)

**DOI:** 10.3389/fphys.2018.00149

**Published:** 2018-02-28

**Authors:** Yan-Dong Jiang, Xin Yuan, Wen-Wu Zhou, Yue-Liang Bai, Gui-Yao Wang, Zeng-Rong Zhu

**Affiliations:** State Key Laboratory of Rice Biology, Key Laboratory of Molecular Biology of Crop Pathogens and Insects, Ministry of Agriculture, Institute of Insect Sciences, Zhejiang University, Hangzhou, China

**Keywords:** light entrainment, circadian clock, cryptochrome, molecular oscillation, behavioral rhythm

## Abstract

Most living organisms have developed internal circadian clocks to anticipate the daily environmental changes. The circadian clocks are composed of several transcriptional-translational feedback loops, in which cryptochromes (CRYs) serve as critical elements. In insects, some CRYs act as photopigments to control circadian photoentrainment, while the others act as transcriptional regulators. We cloned and characterized two *cryptochrome* genes, the *Drosophila*-like (*lscry1*) and vertebrate-like (*lscry2*) genes, in a rice pest *Laodelphax striatellus*. Quantitative real-time PCR showed that *lscry1* and *lscry2* expressed ubiquitously from nymph to adult stages as well as in different tissues. The transcript levels of *lscry2* fluctuated in a circadian manner. Constant light led to arrhythmic locomotor activities in *L. striatellus*. It also inhibited the mRNA oscillation of *lscry2* and promoted the transcription of *lscry1*. Knockdown of *lscry1* or *lscry2* by RNA interference (RNAi) reduced the rhythmicity of *L. striatellus* in constant darkness, but not in light dark cycles. These results suggested that *lscry1* and *lscry2* were putative circadian clock genes of *L. striatellus*, involved in the regulation of locomotor rhythms.

## Introduction

Circadian rhythm is an endogenous cycle for the biochemical, physiological and behavioral processes in living organisms. In insects, the circadian rhythm regulates the behaviors like locomotor activity (Pavan et al., 2016), feeding (Seay and Thummel, 2011; Suszczynska et al., 2017), mating (Fuchikawa et al., 2010), and eclosion (Myers et al., 2003), and facilitates the adaptation of insects to daily environmental changes. The circadian rhythm persists in constant conditions and can be entrained by environment cues, the “Zeitgebers”, including light (Tao et al., 2017), temperature (Tomioka and Matsumoto, 2010), vibration (Simoni et al., 2014), and food availability (Sancar and Brunner, 2014).

In *Drosophila*, light signals regulate the circadian rhythm through its perception by a cell-autonomous circadian photoreceptor cryptochrome (CRY). CRYs, first identified in plants, serve as core components of the circadian clock in animals. Generally, the animal CRYs can be divided into two types: (I) *Drosophila*-like CRYs, which act as non-visual photopigments linking circadian rhythms to the environmental light/dark cycle, and (II) vertebrate-like CRYs which act as transcriptional repressors and control the generation of circadian rhythms. Some insects have only one type of CRY, e.g., type I for fruit flies (Emery et al., 1998), and type II for honeybees (Rubin et al., 2006) and ants (Ingram et al., 2012). But the others have both types, such as monarch butterflies (Zhu et al., 2008) and mosquitoes (Rund et al., 2011). Insects that have lost the function of *cry* displayed a disrupted rhythm of adult emergence (De et al., 2012; Merlin et al., 2013) and an impairment of the visual behavior (Mazzotta et al., 2013). Causal involvement of vertebrate-like *cry* in circadian cuticle deposition was shown in the bean bug *Riptortus pedestris* (Ikeno et al., 2011).

Circadian rhythms are controlled by a multi-clock system: one central clock in the brain organizing the overall behavioral rhythms and several peripheral clocks residing in a variety of peripheral tissues regulating specific physiological rhythms (Tomioka et al., 2012). Most insect circadian clocks are based on the negative feedback loops comprised of several conserved clock genes. These clock genes show diverse roles in different species (Tomioka and Matsumoto, 2015). So far, the central clock system is most well-understood in *Drosophila melanogaster*. It comprises at least three transcriptional-translational feedback loops. One core loop involves the canonical clock genes *period* (*per*), *timeless* (*tim*), *clock* (*clk*), *cycle* (*cyc*), and *cryptochrome* (*cry*) (Crane and Young, 2014). In this loop, the CLK:CYC heterodimer acts as a positive element for the activation of the transcriptions of *per, tim* and other clock genes. PER and TIM form a complex in the dark and bind to the CLK:CYC heterodimer in nucleus, which in turn inhibit the transcriptions of *per* and *tim* (Yuan et al., 2007; Crane and Young, 2014). The CRY protein is stable in the dark and can convert to an unstable conformation in the presence of light. It light-dependently binds to TIM and irreversibly commits TIM to degradation (Busza et al., 2004). Once TIM and PER are degraded, CLK:CYC activity is restored to initiate a new cycle of transcriptions. However, the feedback loop is different in the monarch butterfly in which transcriptions of clock genes are repressed by vertebrate-like CRY via independent repression of CLOCK and BMAL1 activity (Zhu et al., 2008; Merlin et al., 2013; Zhang et al., 2017). In bees and beetles, vertebrate-like CRY proteins function as light-insensitive transcriptional repressors, which propose novel circadian clock mechanisms (Yuan et al., 2007). Recently, high throughput sequencing techniques gave rise to more studies in the characterization of clock genes in non-model insects (Tomioka and Matsumoto, 2015). Beyond the roles in the circadian clocks, CRYs are also suggested to be candidate magnetoreceptors. CRYs played a vital role in the migration of the monarch butterfly by providing precise timing components for sun compass orientation (Zhu et al., 2008). The light activation of CRY mediated the sensitivity of *Drosophila*'s circadian clock to the magnetic fields (Yoshii et al., 2009). Recently, the *cry*-dependent sensitivity to the direction of geomagnetic fields was borne out in two cockroach species using gene silencing and a directional magnetic field (Bazalova et al., 2016).

The small brown planthopper (SBPH) *Laodelphax striatellus* (Fallén) (Hemiptera: Dalphacidae) is an important pest of rice and can also attack a wide range of other crops. It is a migratory pest and causes severe damage by transmitting plant viruses, such as rice stripe virus (RSV) and rice black-streaked dwarf virus (RBSDV). The density of *L. striatellus* in eastern China rapidly increased in early 2000s (Zhu et al., 2005; Liu et al., 2006). Mass migrations of SBPH causing subsequent outbreaks of the rice stripe viral disease have been reported in Japan and Korea since 2008 (Otuka, 2013). The take-off behavior of SBPH in migrations displays a daily rhythm (Sanada-Morimura et al., 2015), but whether it is under the control of circadian clocks remains unclear.

In this study, we analyzed the locomotor activity rhythms of *L. striatellus*, and characterized the sequence and daily expression rhythms of the *cry* genes. The functional examination of the *cry* genes by RNA interference (RNAi) might provide more insights into how they are involved in the determination of the free-running period. These results proved the first understanding of the endogenous clocks of SBPH.

## Methods

### Insects rearing

The strain of *L. striatellus* used in all experiments originated from a field population collected in Hangzhou in eastern China. The insects were reared on susceptible rice seedlings cv. Taichung Native 1 (TN1) at 26 ± 1°C and 60% relative humidity in a 12:12 light:dark (LD) cycle.

### Behavioral analysis

The locomotor activity of SBPH was measured based on a *Drosophila* Activity Monitor 2 (DAM2) system (TriKinetics, USA). Newly emerged adults were individually placed into glass tubes (5 mm diameter ^*^ 65 mm length) with a rice seedling on one side and a small piece of yarn on the other side as a stopper (Figure S1). The rice seedlings served as the food source and were fixed by Parafilm with the roots out of grass tubes. A 2-ml centrifugal tube was used to provide water covering each seedling root. Movements were automatically recorded as infrared beam breaks at 1 min intervals. Monitors were placed in an incubator with environmental conditions kept at 26°C and 60% relative humidity for all experiments. Water in the centrifugal tubes was checked every day and refilled as needed.

The tested SBPH was reared in the incubator under a 12:12 LD photoperiod at nymph stage. The light onset was taken as Zeitgeber time 0 h (ZT0). Newly emerged adults were picked and placed into the monitor tubes for locomotor activity analysis. *L. striatellus* shows wing dimorphism in response to environmental cues, and both macropterous (long-winged) and brachypterous (short-winged) morphs were used. The photoperiod of the adults' first day in tubes was same with that in the incubator because the insects need time to adapt to the condition in tubes. The different light regimes, i.e., LD, constant light (LL) and constant darkness (DD), started at ZT0 of the second day and lasted for at least 4 days. The activity data of the first day was excluded from analyses. The total average activity at 24 h intervals was calculated from 4 days to compare activity levels under different conditions for each individual. Circadian rhythm of locomotor activity was analyzed quantitatively using the Sleep and Circadian Analysis MATLAB Program (SCAMP) (Donelson et al., 2012). Individual sleep plots were used to determine the survival rate of tested adults. Autocorrelation function and maximum entropy spectral analysis (MESA) were used to estimate the periodicity (Levine et al., 2002). Only adults displaying rhythm in both methods were defined as rhythmic individuals. The period of rhythm was calculated by autocorrelation analysis. The height of the third peak in the correlogram was termed as Rhythmicity Index (RI) and indicated the strength of rhythm. Locomotor activity was viewed in actogram format using ActogramJ (Schmid et al., 2011).

### Molecular cloning

Approximately 100 male heads and 100 female heads were pooled together from which total RNA was extracted using TRIzol reagent (Invitrogen, USA) according to the manufacturer's protocol. The RNA was treated with DNase (Takara, Japan) and then reverse transcribed to cDNA using PrimeScript 1st strand cDNA synthesis kit (Takara, Japan).

Previously, one transcriptome dataset for *L. striatellus* was built by Zhang et al. (2010) and another one had been built in our laboratory (unpublished data). Based on the sequence data obtained from transcriptome searching using the basic local alignment search tool (BLAST), gene-specific primers were designed and used for cloning *cry* cDNA (Table S1). PCR were conducted using KOD FX DNA polymerase (Toyobo, Japan). The PCR conditions were determined empirically for the amplification of each gene. PCR products were verified by 1% agarose gel electrophoresis. Target bands were excised and purified using DNA gel extraction kit (Axygen, USA). The DNA fragments were TA ligated into pMD18-T vector (Takara, Japan) and transformed into *Escherichia coli* DH5α (TransGen, China). Positive clones were sequenced by SunnyBio (China).

### Phylogenetic and domain analysis

*L. striatellus* CRYs and their orthologs from other insect species were aligned using Clustal Omega (http://www.ebi.ac.uk/tools/msa/clustalo/). A phylogenetic tree was constructed by MEGA 5 using the neighbor-joining method with 1,000 bootstrap replications. GenBank accession numbers of genes used are listed in Table S2. SMART (Letunic et al., 2015) and CDD (Marchler-Bauer et al., 2015) were used for motif prediction. Nuclear localization signals (NLSs) were predicted by NLStradamus based on an analysis of the residues frequencies of known NLSs using hidden Markov models (HMMs) (Nguyen Ba et al., 2009). Schematic representation of cryptochrome proteins was drawn using Illustrator for Biological Sequences (Liu et al., 2015).

### Gene expression analysis

The concentration of each RNA sample for quantitative real-time PCR (qPCR) was adjusted to 1 μg/μl with nuclease-free water. The RNA was reverse-transcribed in a 20 μl reaction system using ReverTra Ace qPCR RT Kit (Toyobo, Japan). β*-actin* and *glyceraldehyde-3-phosphate dehydrogenase* (*GAPDH*) of *L. striatellus* was used as internal control genes (GenBank accession numbers: AY192151 and HQ385974). A pre-run test was carried out to confirm the constant expression of β*-actin* and *GAPDH* in different samples. The qPCR was performed on a Bio-Rad CFX96 Real-Time System (Bio-Rad Laboratories, USA) using the SYBR Green Realtime PCR Master Mix (Toyobo, Japan) according to the manufacturer's protocol. Primers were designed to determine the relative mRNA expression levels of unique genes (Table S1). Production of the standard and melting curves confirmed the specificity and accuracy of the primers. The threshold cycle values were calculated using three independent biological samples. A non-template control was included for each biological replicate. The 2^−ΔΔCt^ method was used to calculate the relative expression levels (Livak and Schmittgen, 2001).

### Sample collection and light conditions

To investigate transcription profiles at each developmental stage, the first to fifth instar nymphs and adults (from the first to seventh days after emergence) were collected at ZT0. Individuals were immediately frozen in liquid nitrogen and stored at −80°C. Total RNA was extracted from the heads of 30 individuals for each instar. Three biological replicates were used.

For studies of tissue-specific expression, macropterous and brachypterous adults were collected separately at ZT0 and frozen immediately in liquid nitrogen. These samples were dissected into head, thorax, abdomen, legs, and wings and used for RNA extraction. Each sample contained 50 adults and replicated three times.

To investigate circadian rhythmic expression patterns, male *L. striatellus* was collected at 3-h intervals within the 2 days after emergence in LD followed by 1 day in DD. In order to investigate the influence of light regimes at a long time scale, the nymphs were reared in DD or LL from the 2nd instar and used for gene expression analysis after they developed into adults. Total RNA was extracted from their heads. Three biological replicates were used for each time point with 20 individuals in each sample.

### Knockdown of *lscry1* and *lscry2* by RNAi

The 500-bp fragments of *lscry1* and *lscry2* cDNA (Figure S6) were amplified by PCR using primers containing T7 RNA polymerase promoter sequences (Table S1). The PCR products were used as templates for dsRNA synthesis using the MEGA script T7 kit (Ambion, USA). The synthesized dsRNAs were dissolved in ultra-pure water and quantified with a Nanodrop 2000 microspectrophotometer (Thermo Scientific, USA). Their integrity was determined by agarose gel electrophoresis. Green fluorescent protein (GFP) dsRNA was used as a control. Late fifth instar nymphs were selected based on the morphological characters. Approximately 100 ng dsRNA (4 mg/ml) was injected into each nymph, following the protocol described by Wang et al. (2012). Adults that emerged within 48 h after injection were collected and used for bioassays. The transcript levels of circaidian genes in injected adults were checked by qPCR at ZT6 and ZT18.

### Statistical analysis

The one-way analysis of variance (ANOVA) followed by Tukey's *post-hoc* test was used to compare the differences in mRNA relative expression levels among the different times and the differences in circadian rhythm parameters among treatments. To compare the values of the two groups, *t*-test was used. A cosine wave model [gene expression = cos (time/24^*^2π+phase) ^*^ amplitude + shift] was fitted to the data (Tong, 1976; Bertossa et al., 2014).

## Results

### Circadian rhythms of locomotor activity

**Figure 1:**
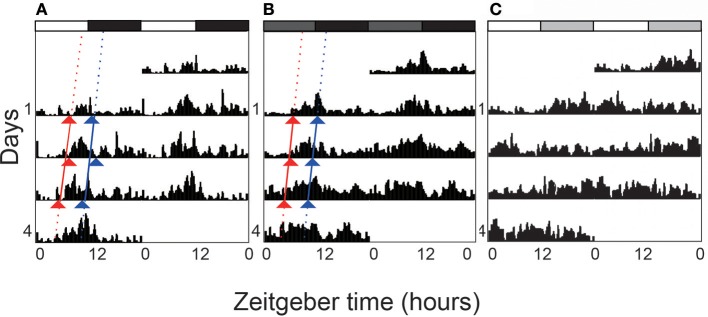
Double-plotted actograms of locomotor activity under different light regimes. *L. striatellus* was reared under 12:12 light:dark (LD) photoperiod during nymph stage and was exposed to LD, constant dark (DD), and constant light (LL) after adult emergence respectively. The actograms show the average activity of tested individuals. In LD **(A)**, white and black bars on the top indicate photophase and scotophase, respectively. Dark gray bars represent “subjective day” in DD **(B)**. Light gray bars represent the “subjective night” in LL **(C)**. Triangles indicate activity onsets (red) and acrophases (blue). The rhythmicity was abolished in constant light.

The newly emerged adults locomoted in a rhythmic pattern during the 4 consecutive days (LD 12:12) (Figure 1A). The locomotor activity kept at a low level in the first half of light phase although the activity counts of the first bins after lights-on were sometime high. Adults became active in the middle of light phase. Major activities were recorded before ZT 12. A drop was recorded after the lights-off followed by low activity in the dark phase. Circadian activity pattern was recorded during the first 3 days in constant darkness (Figure 1B). The activity level was high in the second half of subjective light phase.The rhythm free-ran with a period of ~24 h in the absence of external light/dark cues (Table 1).

**Table 1:** Locomotor rhythms of *L. striatellus* under different light regimes.

**Light regime**	**Rhythmic (%)/*n***	**Total activity per day ± SEM**	**Period ± SEM (h)**	**RI ± SEM**
LD	63.64/33	71.71 ± 7.35	23.75 ± 0.34	0.16 ± 0.02
DD	72.22/18	87.95 ± 15.32	24.08 ± 0.73	0.16 ± 0.09
LL1	40.74/27	51.54 ± 7.98^*^	24.12 ± 0.66	0.11 ± 0.03
LL2	26.67/30	60.44 ± 8.35	23.93 ± 0.96	0.19 ± 0.04

*LD, light:dark 12:12; DD/LL1, the tested individuals were reared under LD during nymphal growth and exposed to constant dark/light at the 1st day after adult emergence; LL2, the individuals were exposed to constant light from the 2nd instar to adult stage. n, sample size*.

* *p < 0.05 compared with LD*.

Light serves as a Zeitgeber to entrain circadian clocks in most organisms. Constant light treatment obviously disturbed the locomotor rhythms. Nearly 60% individuals lost the circadian rhythm on the first day in LL, with the activity counts kept increasing at the subjective night phase (Table 1 and Figure 1C). However, their total locomotor activity per day calculated with 4 days of recording in LL was less than that in LD. For individuals which were entrained to LL from the 2nd instar, the rhythmic proportion reduced to 26.67%. But the period and RI of rhythmic adults were not significantly affected by the constant light treatment.

### Cloning and phylogenetic analysis of *cry* genes

**Figure 2:**
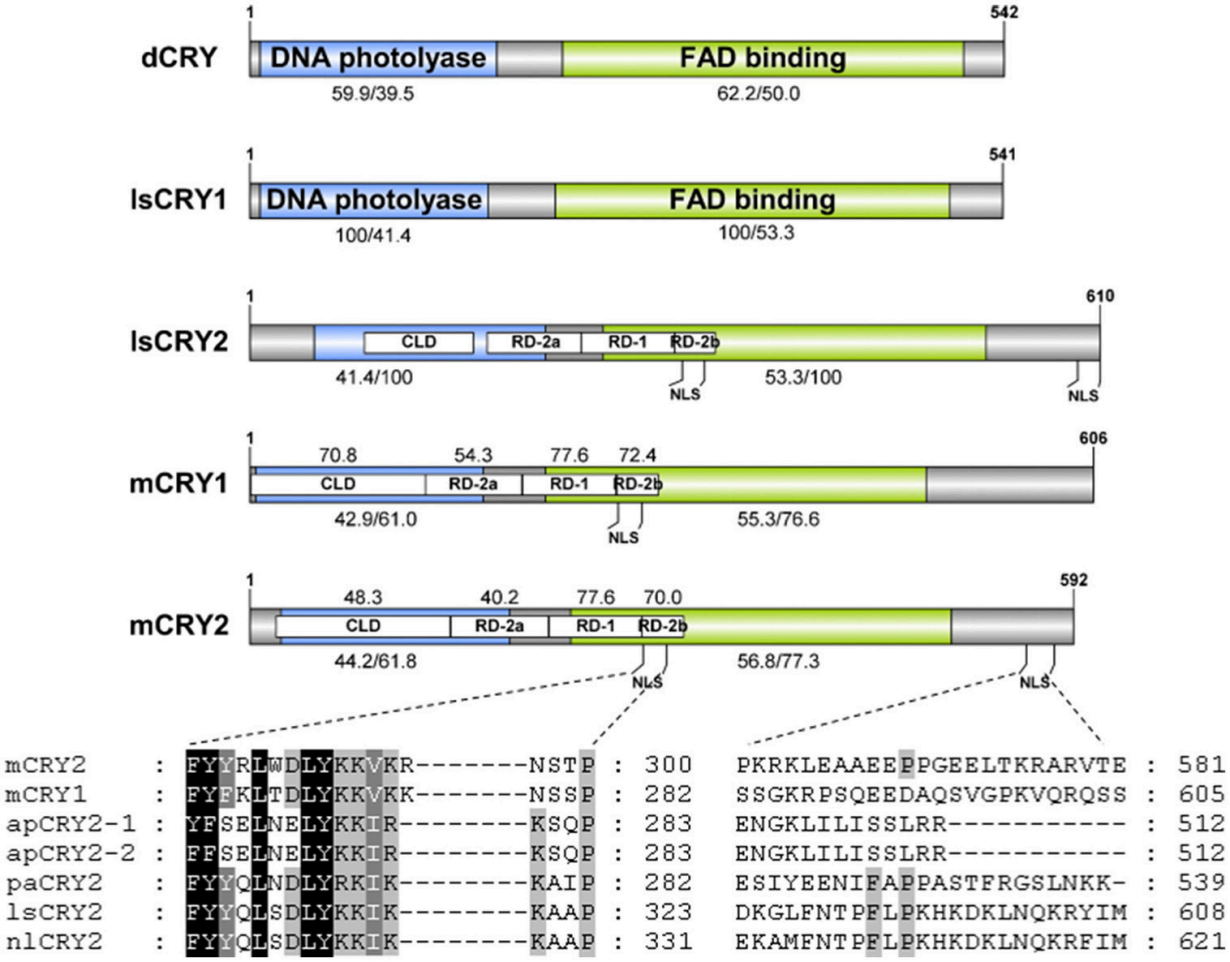
Schematic representation of CRY proteins. Domains were showed including DNA photolyase (blue), FAD binding (green), CLD, RD-2a, RD-1, and RD-2b. Numbers shown under the bar indicate the amino acid identity (%) of DNA photolyase (blue) and FAD binding (green) domain of lsCRY1/lsCRY2 with the orthologs. Numbers above the bar indicate the amino acid identity (%) of CLD, RD-2a, RD-1, and RD-2b domains of lsCRY2 with the orthologs. The number of amino acid residues is shown at the end of each diagram. Low panels show alignments of the putative nuclear localization signals (NLS) in the RD-2b domain (left) and the C-terminus (right) from vertebrate-like CRY proteins (mCRY1, *Mus musculus* CRY1; mCRY2, *M. musculus* CRY2; lsCRY2, *Laodelphax striatellus* CRY2; apCRY2, *Acythosiphon pisum* CRY2; paCRY2, *Pyrrhocoris apterus* CRY2; nlCRY2, *Nilaparvata lugens* CRY2).

**Figure 3:**
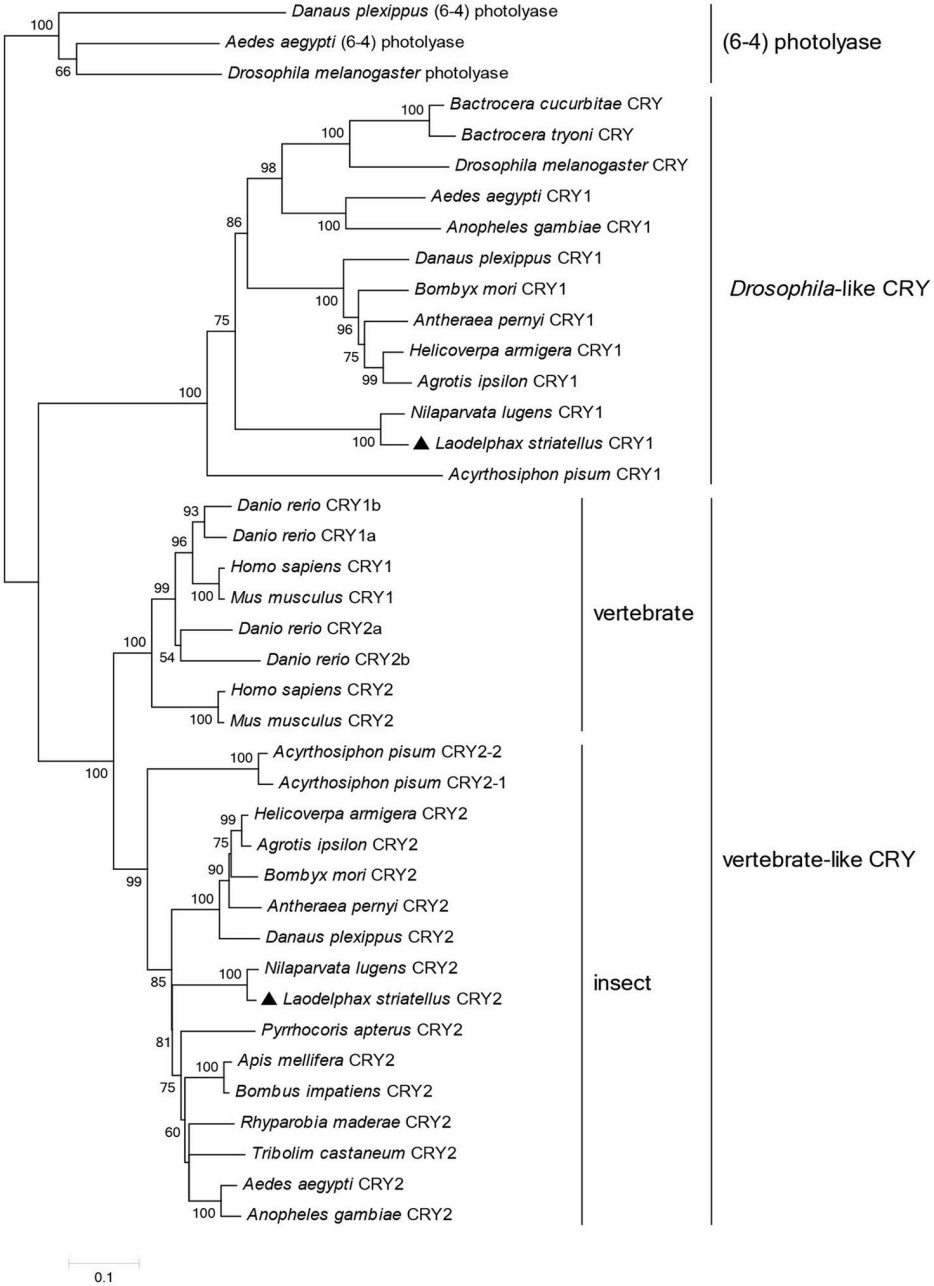
A phylogenetic tree of animal cryptochromes. The tree was constructed using the neighbor-joining method in MEGA5 with (6-4) photolyases serving as an outgroup. Percentages of bootstraps based on 1,000 replicates were indicated with only values >50%. Genbank accession numbers of the sequences are listed in Table S2.

To understand the molecular basis of the photic entrainment of the behavioral rhythms, *cryptochrome* genes were identified in *L. striatellus* based on the transcriptome data. Homologs for both *Drosophila*-like (*lscry1*) and vertebrate-like (*lscry2*) *cry* genes were cloned (GenBank accession numbers: MG356483 and MG356484). *lscry1* encoded a protein of 541 amino acids with an estimated molecular weight of 62.7 kDa. It revealed 56.1% identity when compared with *D. melanogaster*. The deduced lsCRY2 protein had 610 amino acids with an identity of 61.7% to mouse CRY1. The predicted molecular weight was 68.9 kDa. Both lsCRY1 and lsCRY2 had two conserved domains, a DNA photolyase domain and a FAD binding domain. Three conserved domains (RD-2a, RD-1, and RD-2b) and two putative NLSs were found in lsCRY2, but absent in lsCRY1. A cytoplasmic localization domain (CLD) was also found at the N-terminus of lsCRY2 (Figure 2). Phylogenetic analysis of the amino acid sequences of animal CRYs separated lsCRY1 and lsCRY2 into different clades, *Drosophila*-like and vertebrate-like CRYs. Within each clade, *L. striatellus* sequences were most closely related to the brown planthopper *Nilaparvata lugens* homologs, which was consistent with the evolution relationship of these two species (Figure 3).

### Daily transcript levels of *lscry1* and *lscry2*

**Figure 4:**
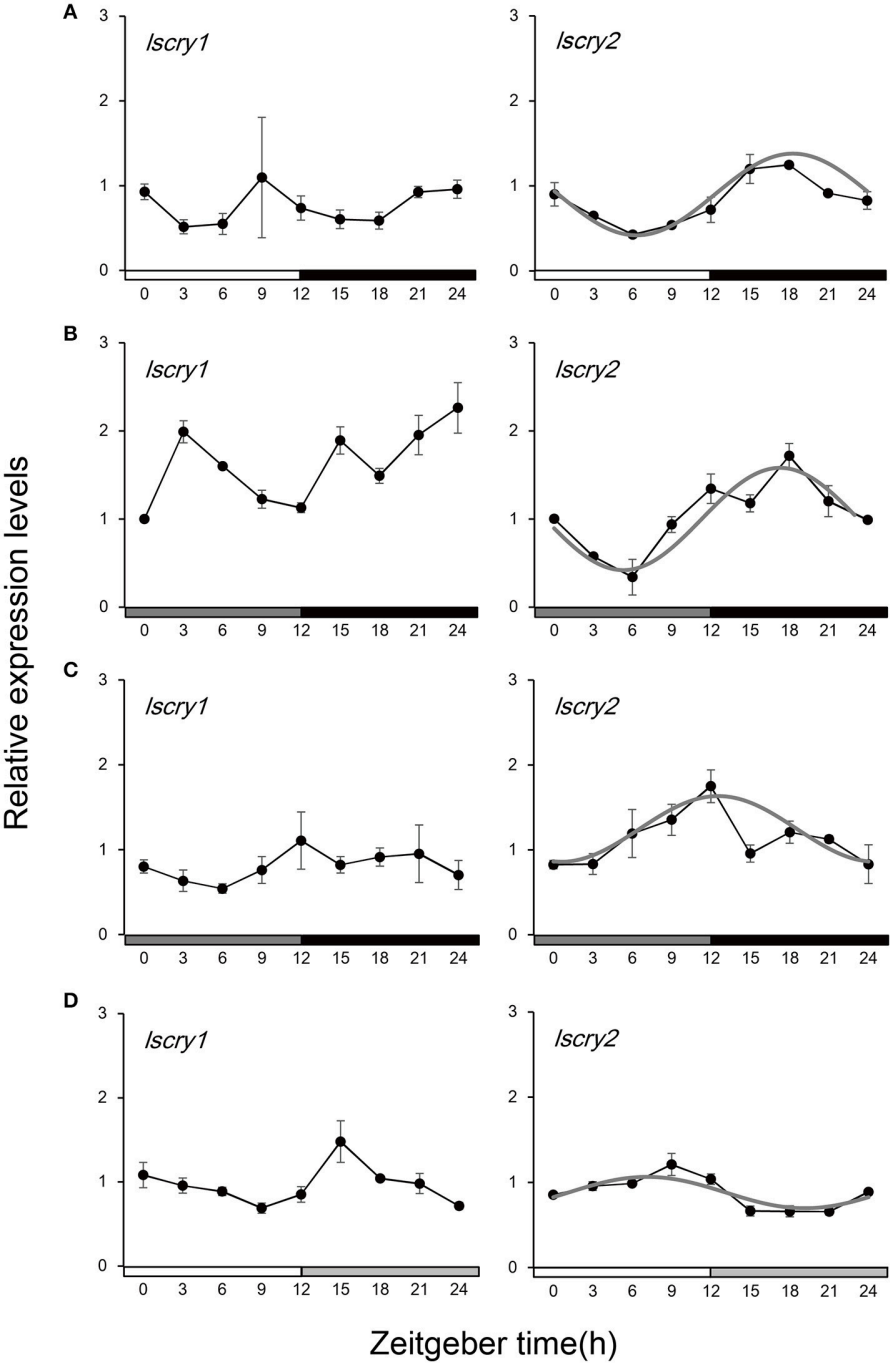
Transcript levels of *lscry1* and *lscry2* in male heads under different light conditions. **(A)** light:dark 12:12; **(B)**1st day in constant dark; **(C)** constant dark treatment; **(D)** constant light treatment. The curves (gray) represent the best cosine wave fit of the experimental data for a fixed period of 24 h. Filled bars on the bottom represent light (white and dark gray) and dark (black and light gray) phases.

Spatio-temporal expression analysis revealed that *lscry1* and *lscry*2 were ubiquitously expressed during the entire developmental stages (Figure S2). In adults, the expression of *lscry1* and *lscry2* mRNA were detected in head, thorax, abdomen, legs, and wings (Figure S3). Daily transcript levels of two *cry* genes in the heads of males were analyzed over the course of a 24-h day. *lscry1* and *lscry2* expressed in different patterns. *lscry1* mRNA constitutively expressed in LD (Figure 4A) and did not show typical circadian rhythms in DD (Figure 4B). A rhythmic pattern of *lscry2* transcript levels were detected with a peak in the middle of dark phase as well as a trough at light phase (Figure 4A). The rhythm of *lscry2* transcript levels persisted in the first day of DD (Figure 4B). Furthermore, the influence of constant dark was also examined in a relative long time scale by placing the insects in DD from the 2nd instar. No significant difference of *lscry1* transcript abundances was detected among Zeitgeber times. But *lscry2* transcript still showed a weakened oscillation in DD with the peak shifted to ZT 12 (Figure 4C).

The transcript levels of *cry* genes were also influenced by the constant light treatment. As shown in Figure 4D, *lscry1* kept a constitutive expression in LL. But the transcript abundance throughout the day was higher than that in LD (Figure S4). Moreover, the oscillation of *lscry2* mRNAwas inhibited.

### Transcript levels of circadian genes after the knockdown of *lscry1* and *lscry2*

**Figure 5:**
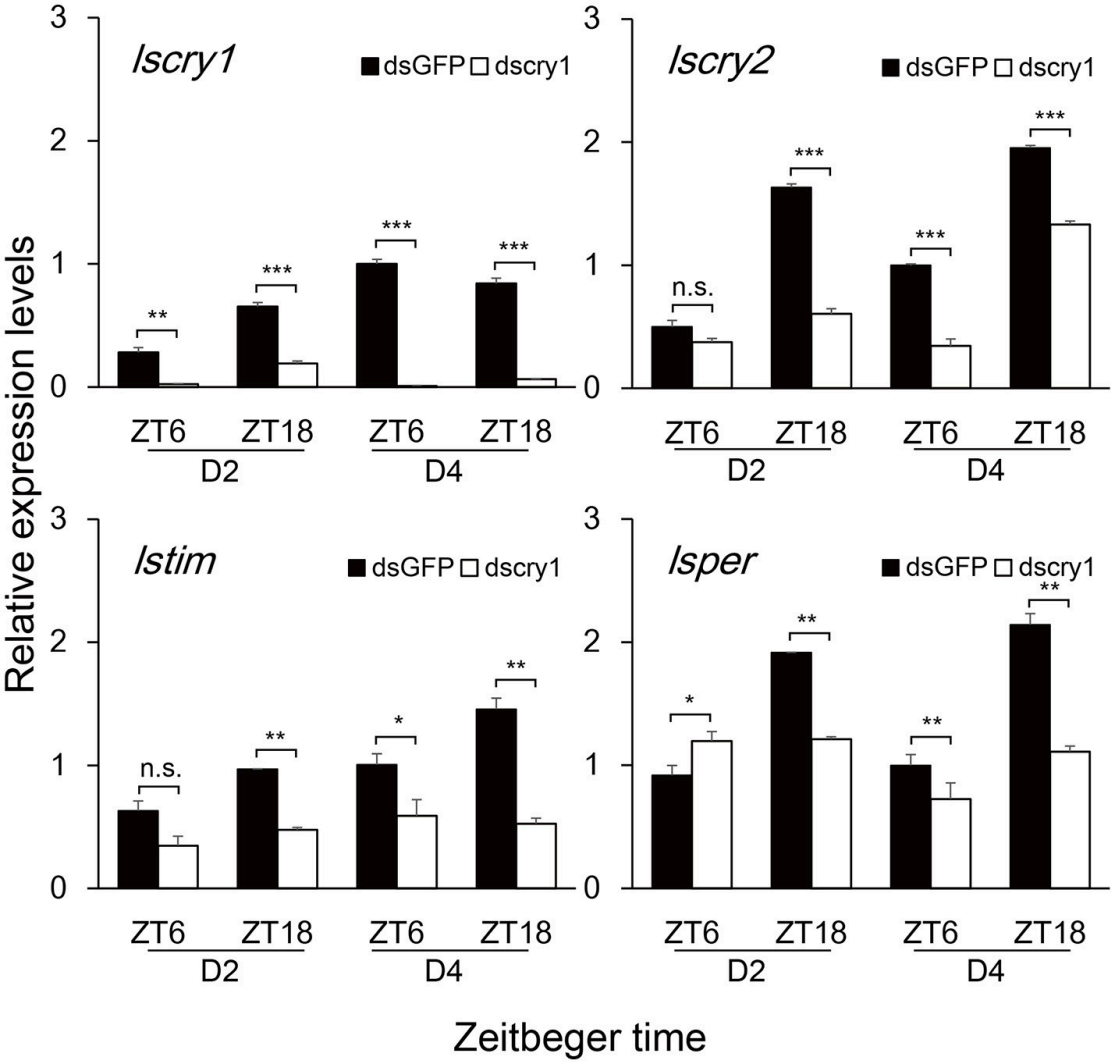
Transcript levels of circadian genes after *lscry1* RNAi. Asterisks indicate significant difference between individuals treated with dsGFP and dscry1 RNA: ^*^P < 0.05, ^**^P < 0.01, ^***^P < 0.001, *t*-test.

**Figure 6:**
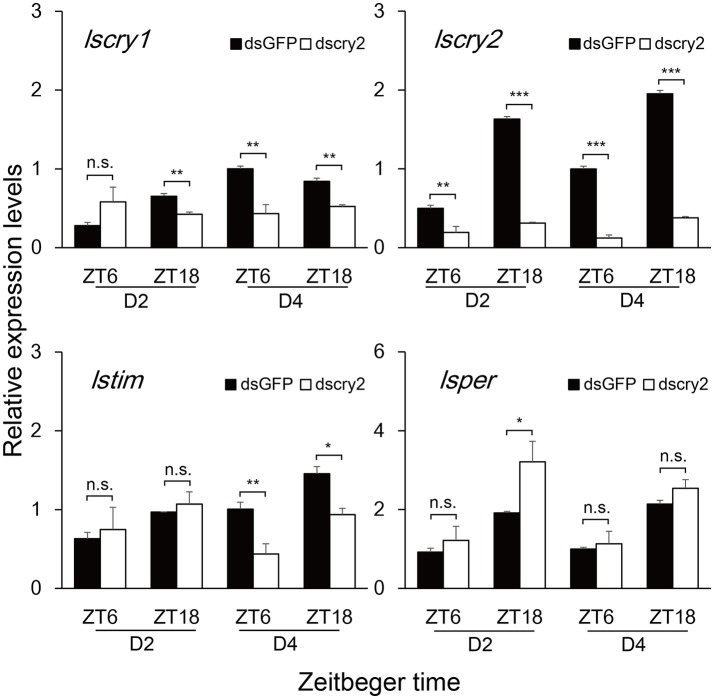
Transcript levels of circadian genes after *lscry2* RNAi. Asterisks indicate significant difference between individuals treated with dsGFP and dscry2 RNA: ^*^P < 0.05, ^**^P < 0.01, ^***^P < 0.001, *t*-test.

To investigate the role of *lscry1* and *lscry2* in the regulation of circadian rhythm, RNAi was performed in the fifth-instar nymphs of *L. striatellus*. Both *lscry1* and *lscry2* displayed a significant decline of transcripts at 48 h after injection, and the effects lasted for 6 days (Figure S5). Furthermore, *timeless* (*lstim*) and *period* (*lsper*) had been cloned in *L. striatellus* and submitted to NCBI before (GenBank No.: MG356486 and MG356485). The transcripts levels of circadian genes were examined to see the effect of *lscry1* and *lscry2* RNAi on the molecular clockwork. Both low and high expression time points (ZT6&ZT18) of *lscry2* were chosen to show the transcript levels dynamically. Knockdown of *lscry1* inhibited the transcript levels of *lscry2, lstim*, and *lsper*, except for ZT6 of the second day after injection (Figure 5). Knockdown of *lscry2* led to a down-regulation of *lscry1* and *lstim* transcript levels on the fourth day after injection. The transcript levels of *lsper* was up-regulated at ZT18 2 days after injection, but was not affected at other time points (Figure 6).

### Locomotor rhythms after the knockdown of *lscry1* and *lscry2*

**Figure 7:**
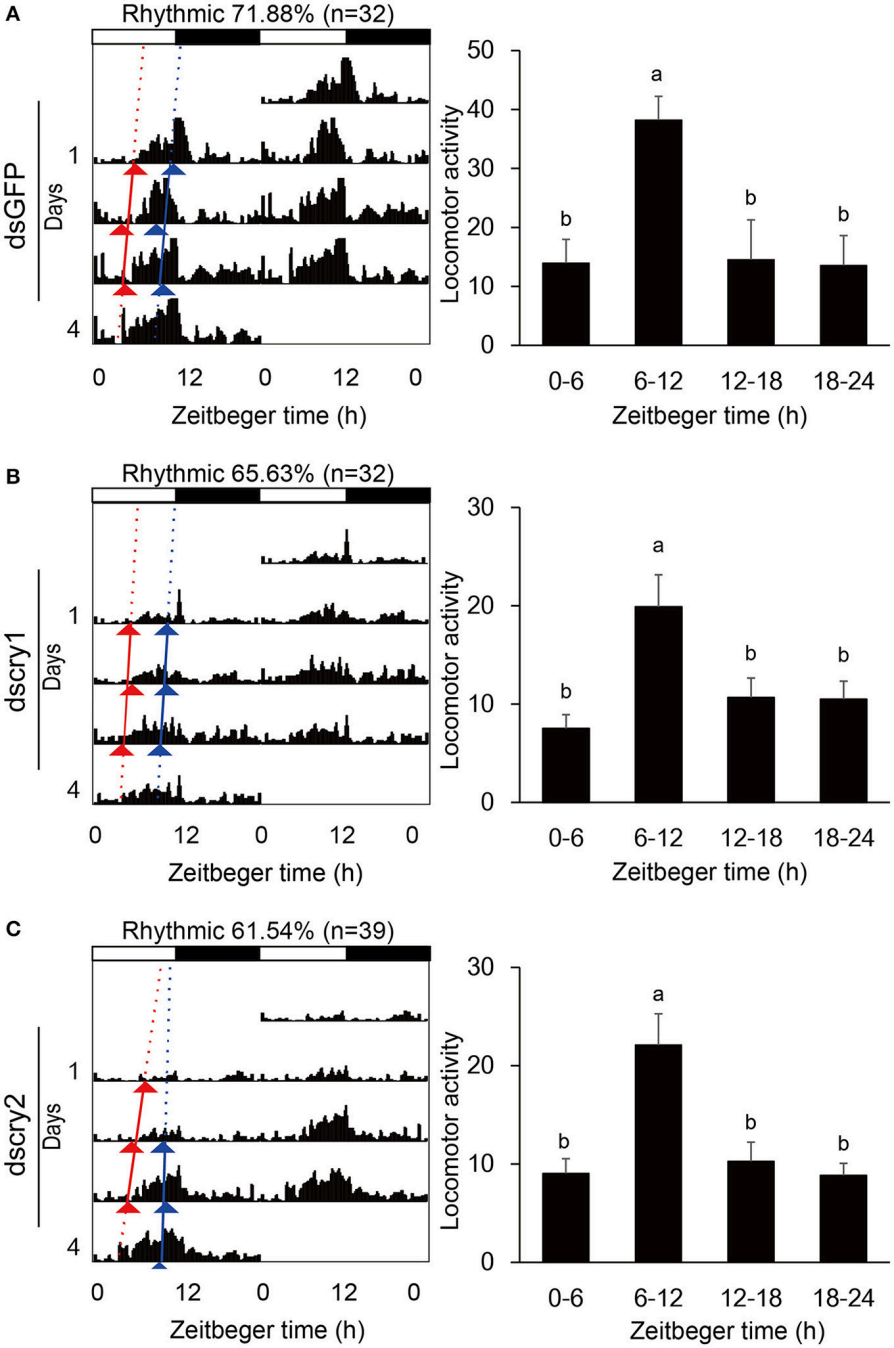
Locomotor activity in LD. Double-plotted actograms (left) and daily activity averages (right) of SBPH injected with dsGFP **(A)**, dscry1 **(B)**, and dscry2 **(C)**. Different letters indicate significant difference among Zeitgeber times (ANOVA followed by Tukey's *post-hoc* test, *P* < 0.05).

**Figure 8:**
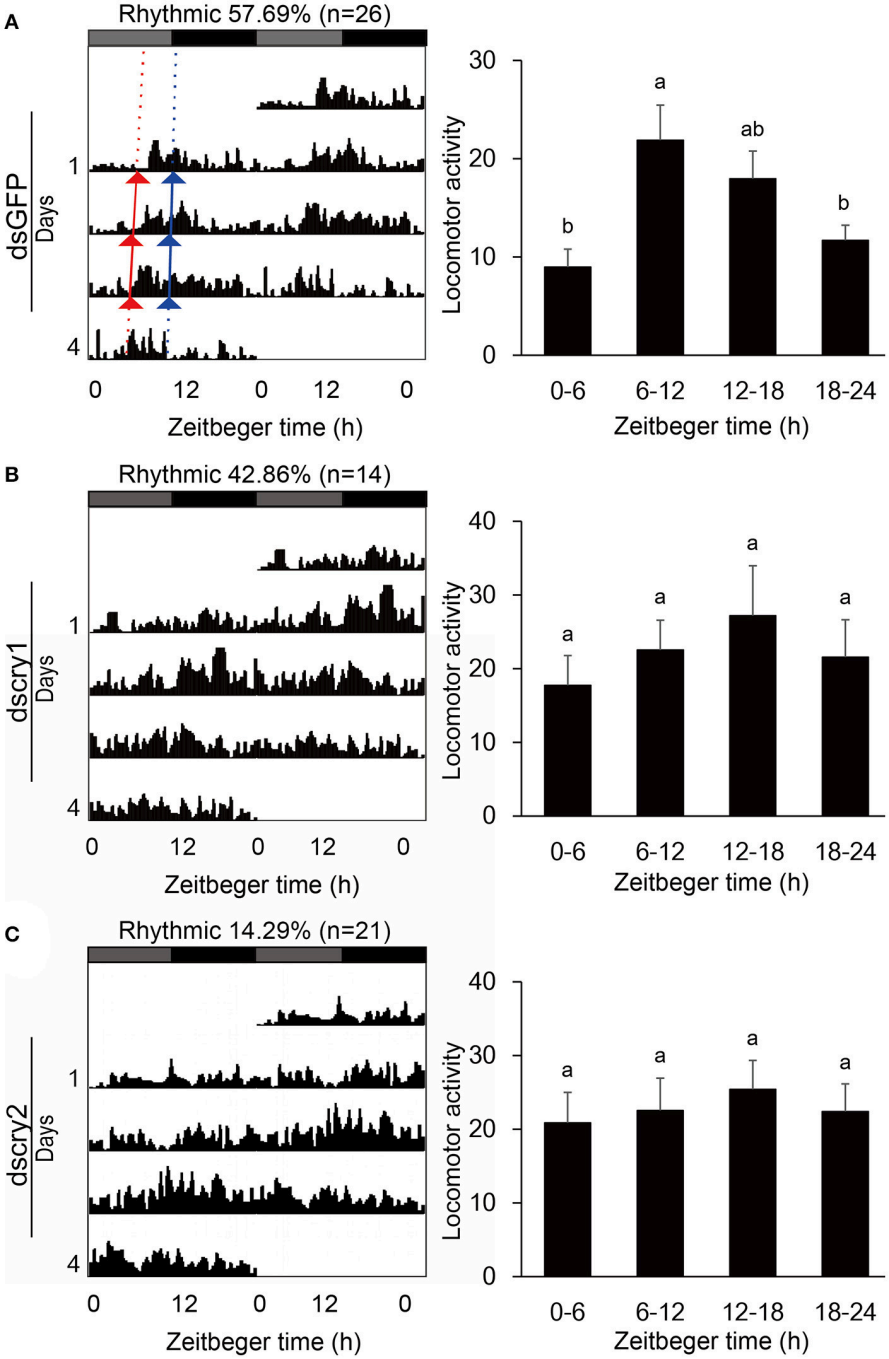
Locomotor activity in DD. Double-plotted actograms (left) and daily activity averages (right) of SBPH injected with dsGFP **(A)**, dscry1 **(B)**, and dscry2 **(C)**. Different letters indicate significant difference among Zeitgeber times (ANOVA followed by Tukey's *post-hoc* test, *P* < 0.05).

Locomotor activity was recorded with the injected adults. As was the case for non-injected ones, the GFP-RNAi, *lscry1*-RNAi, and *lscry2*-RNAi individuals all exhibited a diurnal activity rhythm in LD12:12 with the major peak before lights-off (Figure 7). The average cross counts during ZT6-12 were higher than those of other intervals. Although the *lscry1* and *lscry2* RNAi adults could be entrained to light: dark cycles, their daily activities were significantly decreased (Table 2). However, the circadian locomotor rhythm in DD was strongly influenced by the knockdown of *cry* genes (Table 2 and Figure 8). The percentage of rhythmic *L. striatellus* decreased after the knockdown of *lscry1*. The average counts depicted by values of 4 days showed no significant difference among four intervals. The adults became arrhythmic in DD after the knockdown of *lscry2* with daily activities much higher than the control group.

**Table 2:** Locomotor rhythms of GFP-RNAi, *lscry1*-RNAi and *lscry2*-RNAi individuals.

**Treatment**	**LD**			**DD**		
	**Total activity per day ± SEM**	**Period ± SEM**	**RI ± SEM**	**Total activity per day ± SEM**	**Period ± SEM**	**RI ± SEM**
GFP RNAi	77.66 ± 11.91^a^	23.50 ± 0.36^a^	0.16 ± 0.02^a^	63.49 ± 7.54^b^	23.96 ± 0.36^a^	0.11 ± 0.01^a^
*lscry1* RNAi	48.59 ± 7.01^b^	23.38 ± 0.47^a^	0.17 ± 0.02^a^	51.67 ± 8.91^b^	23.07 ± 1.09^a^	0.10 ± 0.03^a^
*lscry2* RNAi	50.35 ± 6.1^b^	23.71 ± 0.35^a^	0.11 ± 0.02^a^	118.33 ± 17.32^a^	23.97 ± 0.28^a^	0.16 ± 0.02^a^

*LD, light:dark 12:12; DD, constant dark. Different letters indicate significant differences in different treatments (ANOVA followed by Tukey's post-hoc test, P < 0.05)*.

## Discussion

Biological clock serves as an interface in insects' physiology, behavior and the daily light/dark cycles. Although circadian rhythms have been well-documented in *Drosophila*, they are still vague in many non-model insects. Our study is aim to investigate the link between circadian clocks and locomotor rhythms of *L. striatellus*. In this study, we showed a robust and endogenous circadian rhythm of locomotor activity in *L. striatellus* using an automatic monitor system. *Drosophila*-like (*lscry1*) and vertebrate-like (*lscry2*) *cryptochrome* genes were cloned and characterized as the candidate clock genes. Knockdown of *lscry1* and *lscry2* by RNAi disturbed the locomotor rhythms of adults in DD. These results suggested that *cry* genes were involved in the regulation of circadian rhythms in *L. striatellus*.

The rhythm of locomotor activity is one of the most prominent behavioral output of the circadian clock. Here we demonstrate for the first time that the DAM system which is normally used with fruit flies can be applied to analyze the pattern of locomotor activity in *L. striatellus*. It provides a convenient and effective way to monitor the behavioral rhythms of this important pest in the laboratory. According to the DAM data, the locomotor activity of *L. striatellus* was in a diurnal pattern with the major peak appearing before light-off. The slight rising observed after lights-on was more likely to be an instant response to illumination, because the shift from dark phase to light phase was abrupt in the incubator without any gradients of light intensity. Similarly, the masking effects of a lights-on signal were reported on *Drosophila* eclosion (McNabb and Truman, 2008). Although the recording time for *L. striatellus* in our study was shorter than that for *D. melanogaster* due to the shorter life span, it was sufficient to provide an outline of locomotor rhythms. So far, the locomotor activity rhythms of *L. striatellus* have not been reported. But a field investigation demonstrated that the overwintering *L. striatellus* emigrated from wheat fields mainly in the early evening, before the dusk (Sanada-Morimura et al., 2015). Therefore, the major movements of *L. striatellus* we observed in the late daytime are consistent with the migratory habits. However, migration is once in a lifetime and likely to be a response to complex environmental factors and physiological conditions. More studies are needed to investigate whether circadian clock is involved in migration or not. In conclusion, *L. striatellus* had an endogenous circadian rhythm in locomotor activity with major activities occurred in the late light phase.

*L. striatellus* became arrhythmic in the constant light condition, which confirmed that light was an important factor for circadian rhythms. Thus, *cry* were studied as the candidate genes involved in photic entrainment. We hypothesized that the clock mechanism of *L. striatellus* was similar to that of the monarch butterfly in which CRY1 functioned as a blue-light photoreceptor for entrainment, whereas CRY2 functioned as the transcriptional repressor in a negative transcriptional feedback loop. *In-silico* analysis supported the speculation that lsCRY1 and lsCRY2 have divergent roles. RD-2a, RD-1, and RD-2b domains have been proven to be involved in the nuclear translocation and interaction with CLK: BAML1 heterodimer, which are prerequisites for the transcriptional repressor activity (Hirayama et al., 2003). They were highly conserved among repressive-type CRYs. So the presence of these domains as well as NLS and CLD in lsCRY2 suggested that lsCRY2 served as a transcriptional repressor. Notably, besides the NLS in the well-conserved RD-2b region (NLS1), an additional NLS (NLS2) was predicted at the C-terminal extension of lsCRY2. In mammals, NLS in C-terminus is CRY2-specific and important for the nuclear entry of CRY2 and CRY2/PER complex (Khan et al., 2012). In insects, the additional NLS exists in *Apis mellifera* CRY2, but not in *Nasonia vitripennis* CRY or *Danaus plexippus* CRY2 (dpCRY2) (Bertossa et al., 2014). Among all the experimentally verified NLSs, some show characteristic patterns of basic residues loosely matching two consensus sequences, K(K/R)X(K/R) and KRX_10−12_KRXK, whereas some do not match any of the consensus sequences (Nguyen Ba et al., 2009). Although NLS2 in lsCRY2 looks not conserved, it may have some common characteristics in residues frequencies with the known NLSs. It is generally agreed that the C-terminal tails of animal CRYs were quite diverse (Michael et al., 2017). But the exact molecular mechanism of each motif remains unclear. To sum up, although we could not conclude whether the additional NLS made it different from dpCRY2, it is reasonable to infer that lsCRY2 were functional in transcription repression.

The expression of *cry* mRNA under different light regimes were monitored in many insects to understand CRY function in the photic circadian clock. *Drosophila cry* (*dcry*) cycled with a peak in the light phase and a trough in the dark phase (Emery et al., 1998). As to insects which only have vertebrate-like *cry*, such as honeybee and cockroach, *cry* oscillated in an opposite phase with a trough in the daytime and a peak in the night (Rubin et al., 2006; Werckenthin et al., 2012). Lepidoptera insects displayed a combination, in which *cry1* and *cry2* oscillated in a way similar to *dcry* and honeybee *cry*, respectively (Zhu et al., 2008; Yan et al., 2013). In *L. striatellus, lscry2* mRNA oscillated in a circadian way under LD 12:12 with a peak in the late night. The rhythms persisted in the constant darkness. However, *lscry1* mRNA was in a constant level. This result was different from the Lepidoptera models, but consistent with what have been observed in Orthopteran species *Gryllus bimaculatus* (Michael et al., 2017) and two Hemipteran insects *N. lugens* (Xu et al., 2016) and *Acythosiphon pisum* (Cortés et al., 2010). It implied that molecular models of circadian clock established in *D. melanogaster* and *D. plexippus* were not applicable for the clock networks of all insects.

The oscillation of *cry* has also been detected in peripheral clocks located in antennae, compound eyes, malpighian tubules, and reproductive system of *D. melanogaster* (Xu et al., 2008; Yoshii et al., 2008; Tomioka et al., 2012). As expected, the transcripts of *lscry1* and *lscry*2 were detected in multiple peripheral tissues of *L. striatellus* in our study, which implied the existence of peripheral clocks in *L. striatellus* adults. However, with the lacking of the circadian expression profiles of clock genes in peripheral tissues, we could not exclude non-circadian functions of *lscrys*. The diapause vs. reproductive states of the gut in *Pyrrhocoris apterus* was controlled by juvenile hormone through circadian proteins, in which vertebrate-like CRY2 was involved (Bajgar et al., 2013a,b). Wan et al. (2016) suggested that the near-zero magnetic field would affect the positive phototaxis and flight capacity of the brown planthopper via CRYs. Accordingly, it would be interesting to explore the roles of CRYs beyond circadian clocks in *L. striatellus*. It was also not surprising that *lscry1* and *lscry*2 expressed abundantly from 1st instar to adults due to the importance of circadian clocks in life. Higher expression levels in nymphs than those in adults might result from the immaturity of other photoreceptors, since *cry* was not the only receptor for photic signals in most organisms.

Light-induced changes in *cry* gene expression as well as arrhythmic locomotor activity suggested a close connection between *cry* genes and locomotor behaviors. RNAi has proven to be effective in gene silencing and is a convenient and dominant tool to discover the function of novel genes. *lscry1* RNAi individuals synchronized with the given light dark cycle as robustly as the control. This result together with the expression profile suggested that *lscry1* is not the only photoreceptor for entrainment of the clock. *cry* knock-out flies exhibit solid periodicity in DD (Dolezelova et al., 2007). But the crickets treated with dscry1 and dscry2 simultaneously showed a wider range of free-running period than *cry2* RNAi crickets (Tokuoka et al., 2017). In our study, knockdown of *lscry1* reduced the rhythmicity of SBPH in DD. It might be due to the down-regulation of other circadian genes induced by *lscry1* RNAi. As hypothesized in the monarch butterfly, light-induced conformational change in CRY1 make it available to bind with TIM, thus initiating the degradation of TIM. Therefore TIM: PER: CRY2 complex is stable when lacking CRY1, and inhibit the transcription of clock genes by combination with trans-acting element CLK: BMAL1 complex. It might be a reason for the lower transcript levels of other circadian genes in *lscry1* RNAi individuals. The knockdown of *lscry2* led to arrhythmic locomotor activity in DD. It is consistent with studies in crickets (Tokuoka et al., 2017) and cockroaches (Bazalova et al., 2016), in which knockdown of vertebrate-like *cry* led to the disorder of circadian activity. RNAi of *lscry2* also affected the transcript levels of *lsper* and *lstim*. However, the results are time-varying. More time points for sampling are needed to get a thorough understanding of *lsper* and *lstim* mRNA levels. Moreover, the locomotor activity did not change much by *lscry2* RNAi in LD. Knockdown of one gene was not sufficient to disrupt the entire circadian clock network in which several loops might exist providing some compensatory mechanisms. Functional examinations of more circadian genes are desired to get a better understanding of the circadian clock network in *L. striatellus*.

In conclusion, *L. striatellus* exhibited robust circadian rhythms in locomotor activity. Both *Drosophila*-like (*lscry1*) and vertebrate-like (*lscry2*) *cryptochrome* genes were cloned and characterized in *L. striatellus*, which supported the earlier evidence of different clockwork mechanisms in insects. *lscry2* expressed in a circadian manner and participated in the persistence of locomotor rhythms under constant darkness indicating that it might be a core element of the endogenous oscillator. The work provided evidence of the circadian rhythms in *L. striatellus* at both molecular and behavioral levels and enriched the understanding of insect circadian clocks.

## Author contributions

Z-RZ and W-WZ: Conceived and designed the experimental plan; Y-DJ, XY, G-YW, and Y-LB: Performed the experiments; Y-DJ and XY: Analyzed the data and drafted the manuscript.

### Conflict of interest statement

The authors declare that the research was conducted in the absence of any commercial or financial relationships that could be construed as a potential conflict of interest.
