# Supplementary material for: Cryptochrome Regulates Circadian Locomotor Rhythms in the Small Brown Planthopper Laodelphax striatellus (Fallén)
Source: Front Physiol. 2018 Feb 28;9:149. doi: 10.3389/fphys.2018.00149 (PMC5835671; doi:10.3389/fphys.2018.00149)
Supplement: Supplementary file 1 [file DataSheet1.docx]

Supplementary Material

***cryptochrome* regulates circadian locomotor rhythms in the small brown planthopper *L******aodelphax striatellus* (Fallén)**

**Yan-Dong Jiang#, Xin Yuan#, Wen-Wu Zhou, Yue-Liang Bai, Gui-Yao Wang, Zeng-Rong Zhu***

*** Correspondence:** Zeng-Rong Zhu: [zrzhu@zju.edu.cn](mailto:zrzhu@zju.edu.cn)

# These authors contributed equally to this work.

# Supplementary Figures


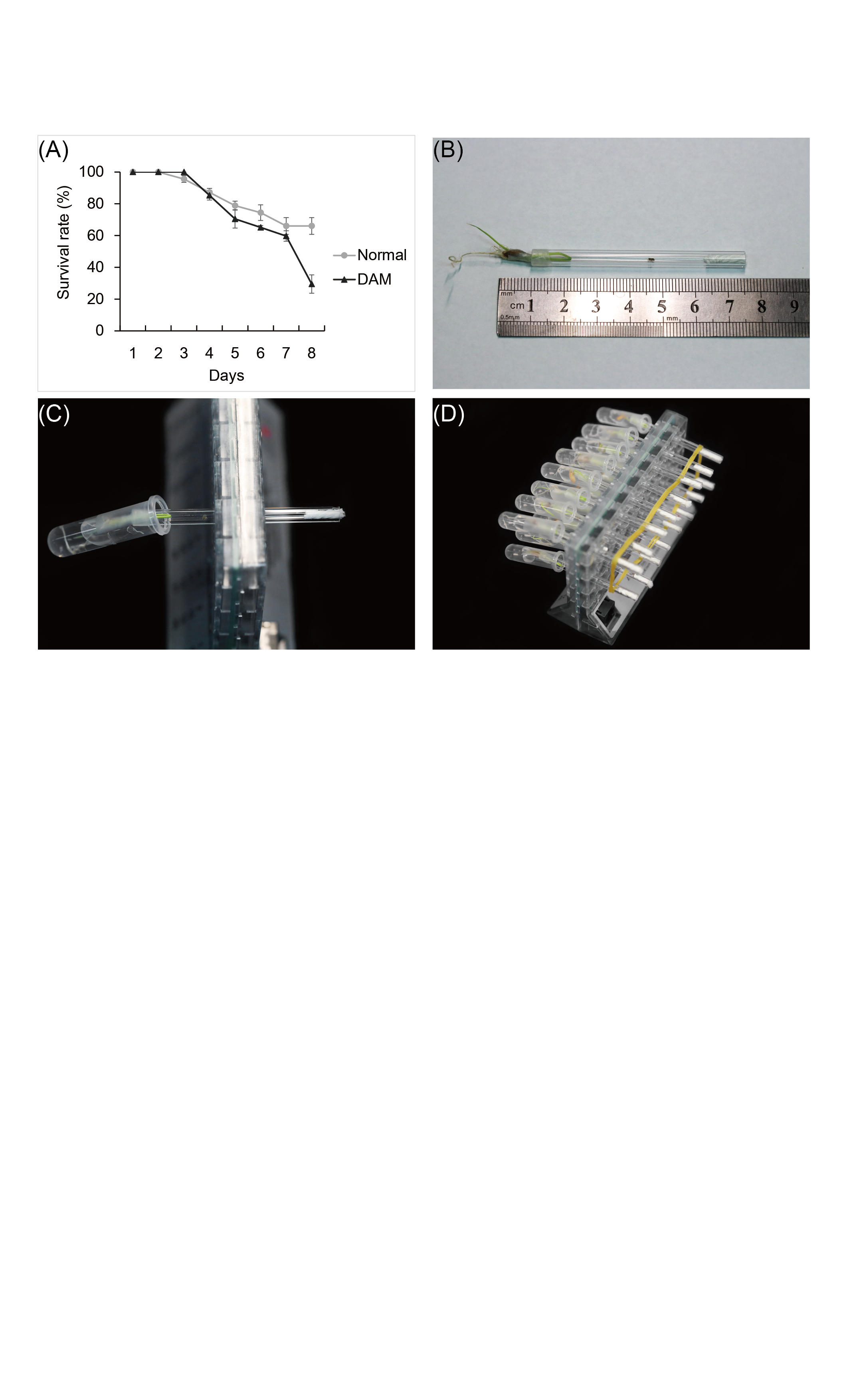


**Supplementary Figure 1.** Locomotor activity monitoring system. (A) Survival rate of *L. striatellus* adults in DAM monitor tubes and normal condition. Data from Day2 to Day6 is used for circadian rhythm analysis. (B) A monitor tube containing a *L. striatellus* adult. A rice seedling is placed into one end of the tube to sustain the insect. A small piece of yarn is placed into the other end. (C) DAM2 monitor contains 32 channels, each measuring the individual activity of an adult in a separate tube*.* A 2-ml centrifugal tube was used to provide water. (D) Fully assembled locomotor activity monitor and tubes. Tubes are fixed by a rubber band to prevent sliding.


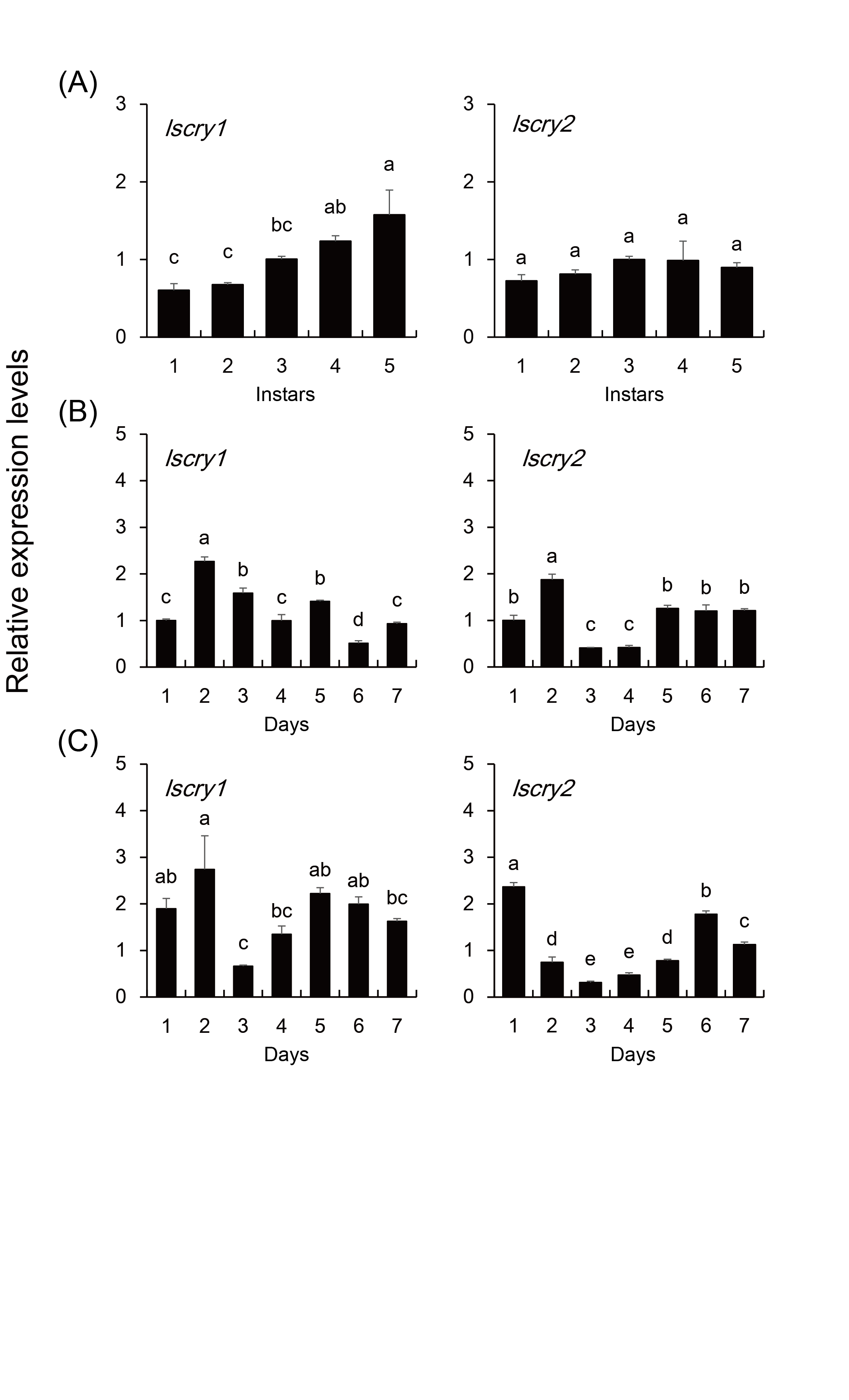


**Supplementary Figure 2.** Relative expression levels of *lscry1* and *lscry2* mRNA at different developmental stages. (A) nymphs in different instars; (B) females in different days after emergence; (C) males in different days after emergence. The data collected from three replications were averaged and plotted as mean ± SEM with the third instar (A) and the first day after emergence (B, C) as the calibrator respectively. Different letters above each bar indicate significant differences (*P* < 0.05).


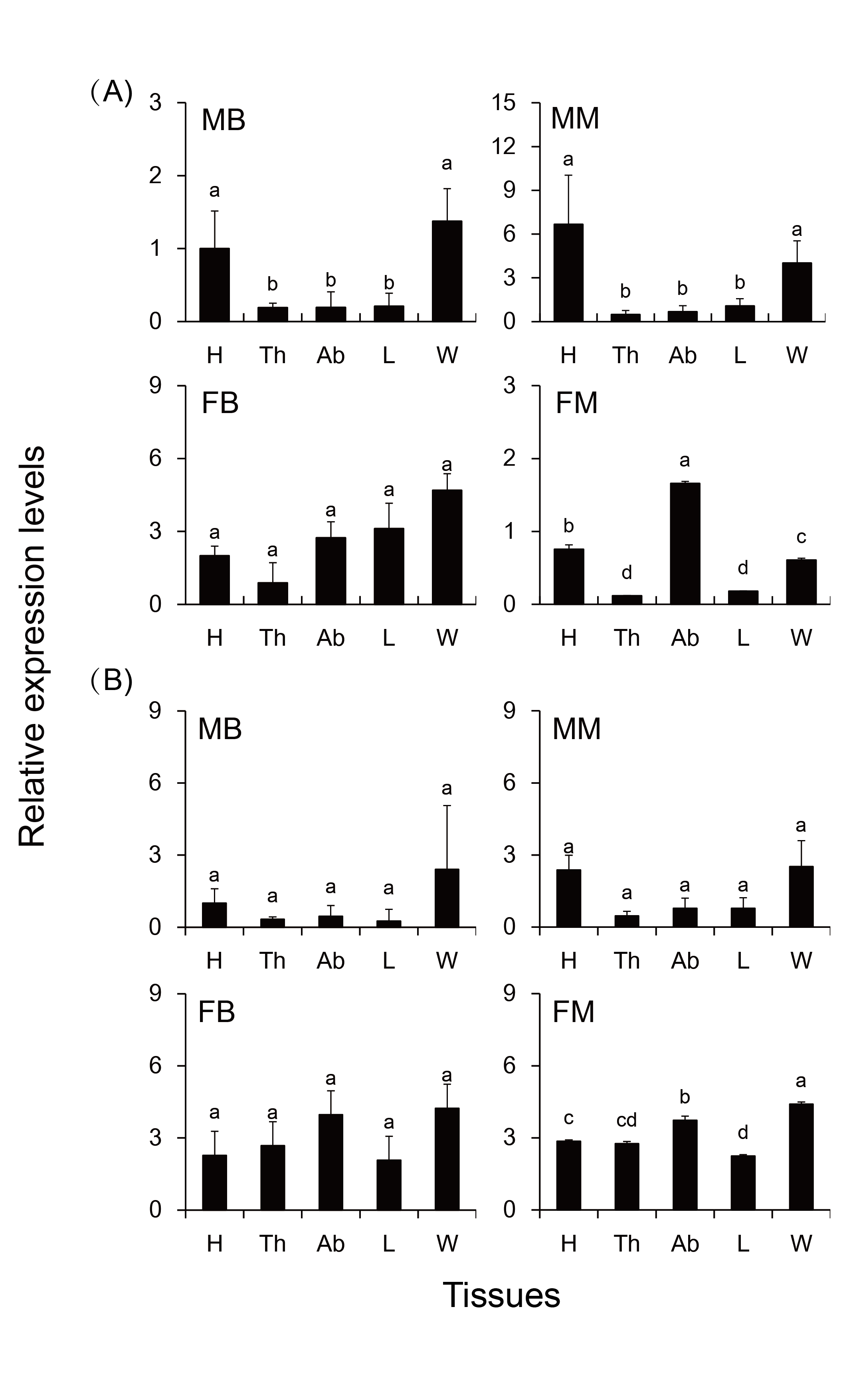


**Supplementary Figure 3.** Relative expression levels of *lscry1* (A) and *lscry2* (B) mRNA in different tissues. H, head; Th, thorax; Ab, abdomen; L, legs; W, wings. MB, brachypterous male; MM, macropterous male; FB, brachypterous female; FM, macropterous female. The mRNA relative expression levels in MB head were set as the calibrators. Different letters above each bar indicate significant differences (*P* < 0.05).


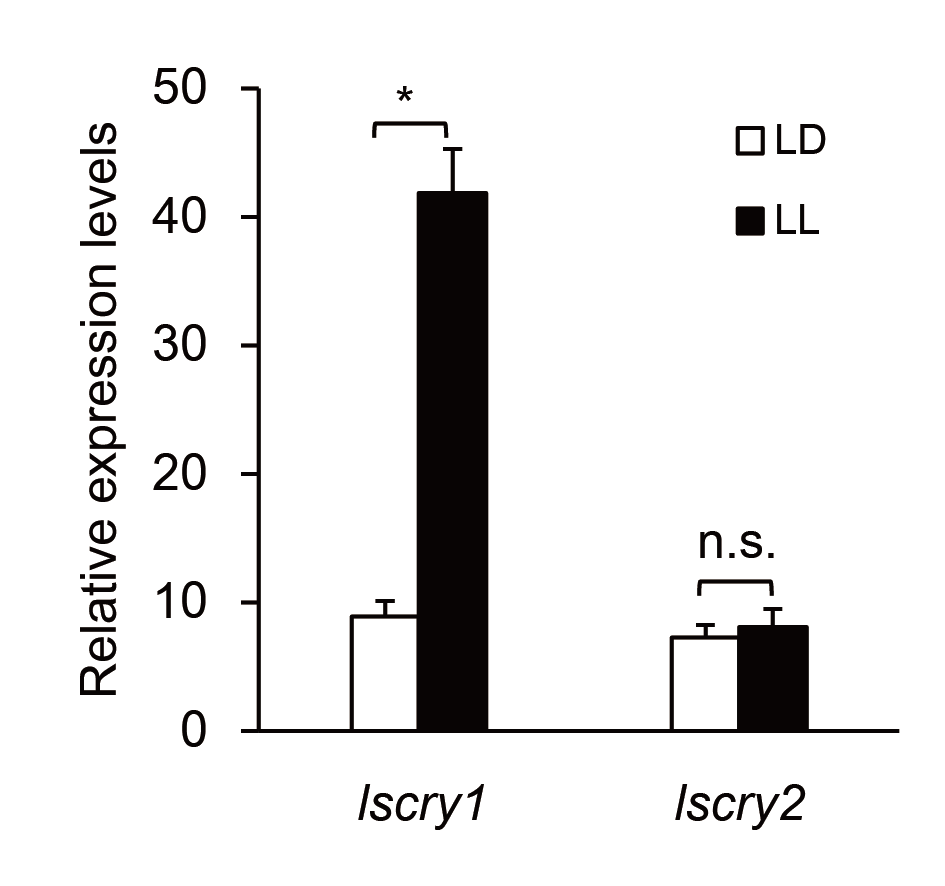


**Supplementary Figure 4.** Daily relative expression levels of *lscry1* and *lscry2* mRNA in different light regimes. LD, light: dark 12:12; LL, constant light. Daily level was the sum of the values of 9 time points in 24 hours. The expression level at ZT0 in LD was calculated as 1. Asterisk (*) indicates significant difference (*P* < 0.05); n.s., not significant.


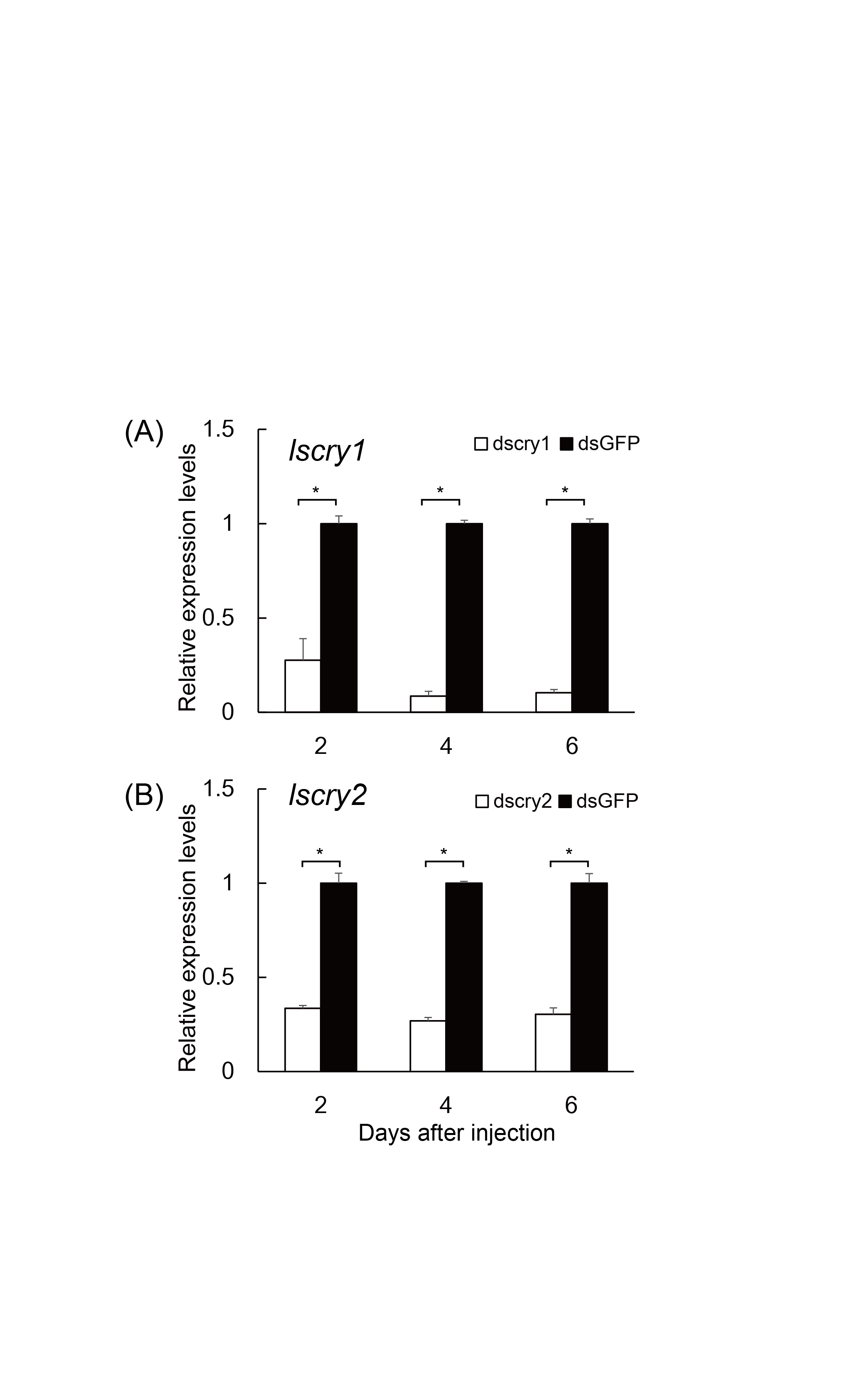


**Supplementary Figure 5.** Effect of *lscry1* RNAi and *lscry2* RNAi on *Laodelphax striatellus*. (a) Relative expression levels of *lscry1* after dscry1 injection; (b) Relative expression levels of *lscry2* after dscry2 injection. Asterisks (*) indicate significant differences (*P* < 0.05).


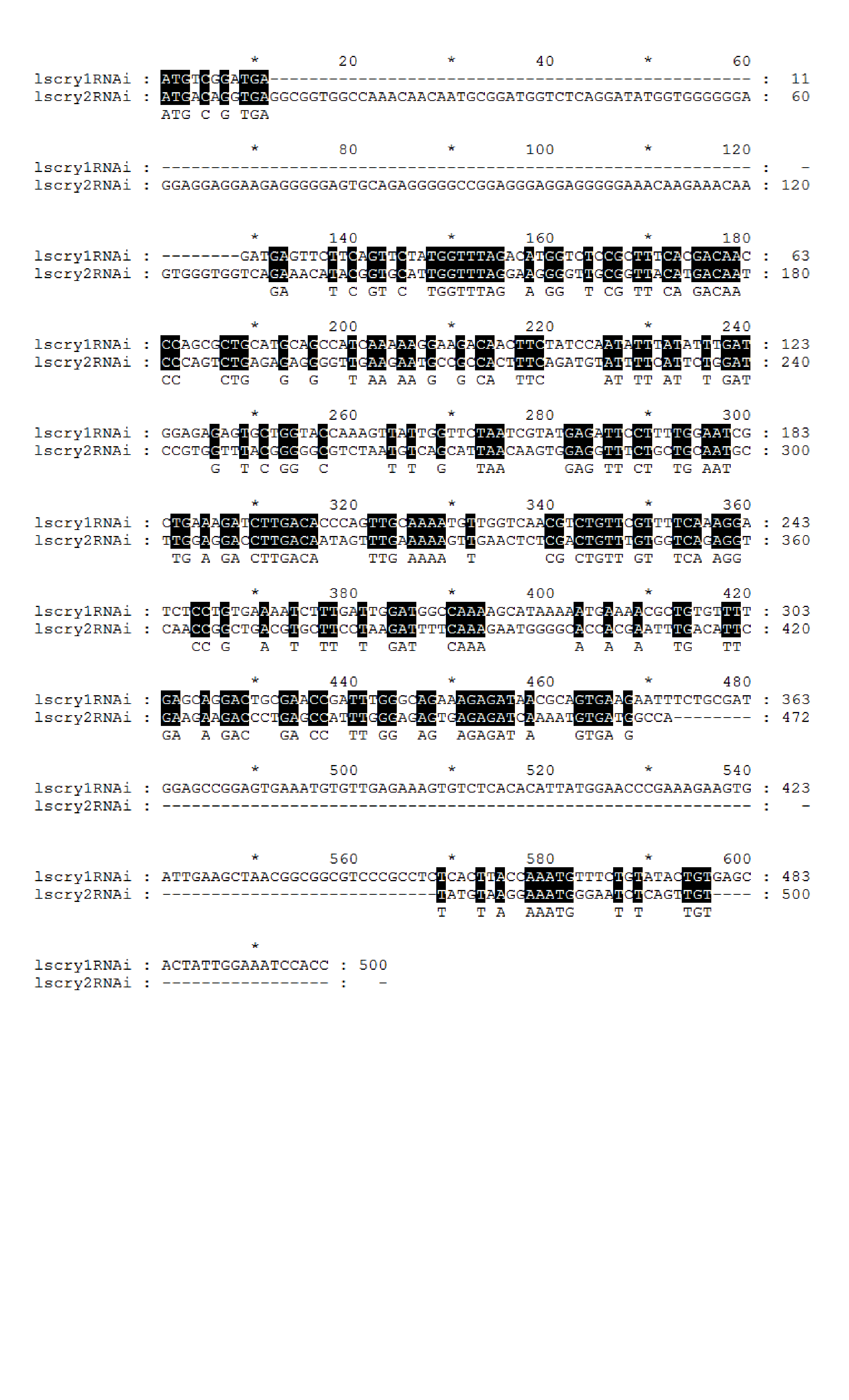


**Supplementary Figure 6.** Alignment of the sequences selected for *lscry1* and *lscry2* RNAi*.*

# Supplementary Tables

**Supplementary Table1.** Primers for gene clone, dsRNA synthesis and qPCR

| Primer Name | Sequence（5’-3’） | Description |
| --- | --- | --- |
| LSCry1_F | GCTACTGGATTCAAATCATTTTC | gene clone |
| LSCry1_R | CTGGCTATTACAAGGTACA | gene clone |
| LSCry2_F | GCGTGCTATGACAGGTGAG | gene clone |
| LSCry2_R | TCACTTGTGCATAATGTAGC | gene clone |
| LSCRY1RNAi_F | GGATCCTAATACGACTCACTATAGGGAGAATGTCGGATGAGATGAGTTC | dsRNA synthesis |
| lscry1RNAi_R | GGATCCTAATACGACTCACTATAGGGAGAGGTGGATTTCCAATAGTGCT | dsRNA synthesis |
| lsCRY2RNAi _F | GGATCCTAATACGACTCACTATAGGGAGAATGACAGGTGAGGCGGTGGC | dsRNA synthesis |
| LScry2RNAi_R | GGATCCTAATACGACTCACTATAGGGAGAACAACTGAGATTCCCATTTC | dsRNA synthesis |
| lsCryRT1_F | ACATCTGCTGGATGCGGATT | qPCR |
| lsCryRT1_R | CTGGGCAGACACAGTGTGAA | qPCR |
| lsCryRT2_F | CTGCTGCTGGATGCTGATTGGT | qPCR |
| lsCryRT2_R | CGGACCGGACAGTAGACATGGA | qPCR |
| LSPER_F | TGGGTCGGATGAGTCGAATG | qPCR |
| LSPER_R | GTCTTCAGTGGGCGACAGTT | qPCR |
| LSTIm_F | GCACTTGCCGTCCGATTCT | qPCR |
| LSTIM_R | TTTCATGGGCCCAAGCTTCT | qPCR |
| lsGAPDH_F | GTACGACTCCACTCACGGAC | qPCR |
| lsGAPDH_R | GTCGCGCTCACTGAATACCT | qPCR |
| lsActin_F | AAACTGGGACGACATGGAGAA | qPCR |
| lsActin_R | GCGACTCGCAACTCGTTGTA | qPCR |

**Supplementary Table2.**Accession numbers of cryptochromes and photolyases

| **Organism** | **Protein** | **Accession Number** |
| --- | --- | --- |
| *Aedes aegypti* | 6-4 photolyase | XP_001658195 |
|  | CRY1 | XP_001648498 |
|  | CRY2 | XP_001655778 |
| *Acyrthosiphon pisum* | CRY1 | NP_001164532 |
|  | CRY2-1 | NP_001164572 |
|  | CRY2-2 | NP_001164573 |
| *Apis mellifera* | CRY2 | NP_001077099 |
| *Agrotis ipsilon* | CRY1 | AFJ22638 |
|  | CRY2 | AFJ22639 |
| *Anopheles gambiae* | CRY1 | ABB29886 |
|  | CRY2 | ABB29887 |
| *Antheraea pernyi* | CRY1 | AAK11644 |
|  | CRY2 | ABO38435 |
| *Bombus impatiens* | CRY2 | ABO31112 |
| *Bactrocera tryoni* | CRY | AAU14170 |
| *Bactrocera cucurbitae* | CRY | BAI67363 |
| *Bombyx mori* | CRY1 | NP_001182628 |
|  | CRY2 | NP_001182627 |
| *Danaus plexippus* | 6-4 photolyase | ABO38436 |
|  | CRY1 | AAX58599 |
|  | CRY2 | ABA62409 |
| *Danio rerio* | 6-4 Photolyase | BAA96852 |
|  | CRY1a | NP_001070765 |
|  | CRY1b | BAA96847 |
|  | CRY2a | BAA96848 |
|  | CRY2b | NP_571867 |
| *Drosophila melanogaster* | 6-4 Photolyase | BAA12067 |
|  | CRY | NP_732407 |
| *Homo sapiens* | CRY1 | AAH30519 |
|  | CRY2 | AAH41814 |
| *Mus musculus* | CRY1 | EDL21416 |
|  | CRY2 | AAD46561 |
| *Nilaparvata lugens* | CRY1 | AJY53623 |
|  | CRY2 | AJY53622 |
| *Helicoverpa armigera* | CRY1 | AEX49898 |
|  | CRY2 | ADN94465 |
| *Pyrrhocoris apterus* | CRY2 | AGI17567 |
| *Rhyparobia maderae* | CRY2 | AGA01579 |
| *Tribolium castaneum* | CRY2 | NP_001076794 |
